# 156. A Quality Improvement Pilot to Increase Hepatitis B Screening and Optimize Patient Selection for Switch to Two-Drug Antiretroviral Regimens in Patients with HIV

**DOI:** 10.1093/ofid/ofae631.042

**Published:** 2025-01-29

**Authors:** Melissa Margolis, Shilpa Vasishta, Judith A Aberg, Antonio E Urbina, Douglas Dieterich, Michael Mullen, Rachel Chasan

**Affiliations:** Icahn School of Medicine at Mount Sinai, New York, NY; Montefiore Medical Center, New York, New York; Icahn School of Medicine at Mount Sinai, New York, NY; Mount Sinai Hospital, New York, New York; Mount Sinai Healthcare System, New York, New York; Icahn School of Medicine at Mount Sinai, New York, NY; The Mount Sinai Hospital, New York, New York

## Abstract

**Background:**

Two-drug antiretroviral regimens (2DR) without tenofovir for HIV are increasingly prescribed. However, these regimens are inadequate for prevention and treatment of hepatitis B virus (HBV). An electronic health record (EHR)-based quality improvement (QI) project was developed to optimize selection of appropriate persons for 2DR within HIV primary care clinics in a New York City health system.

**Figure 1:**
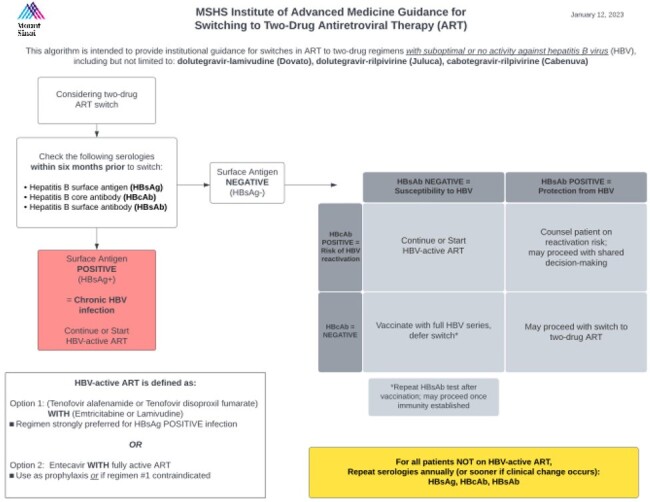
Institutional guidance for switching to two-drug antiretroviral therapy

**Methods:**

An EHR-based ordering flag was implemented on 2DR orders (dolutegravir-lamivudine (DTG-3TC), dolutegravir-rilpivirine (DTG-RPV), cabotegravir-rilpivirine (CAB-RPV)) reminding clinicians to check HBV serologies prior to switch and providing guidance for prescribing 2DR to prevent HBV-related events (infection, relapse or reactivation). Practice patterns were monitored before and after implementation of this QI intervention, including number of individuals screened for HBV (surface antigen (sAg), core antibody (cAb), and surface antibody (sAb)). The analysis compared rates amongst a composite group of individuals with sAg+ (considered at the highest risk) or isolated cAb+ (intermediate-high risk). Outcomes were compared for the one-year periods before and after QI implementation (2022-2023 vs. 2023-2024).

**Figure 2:**
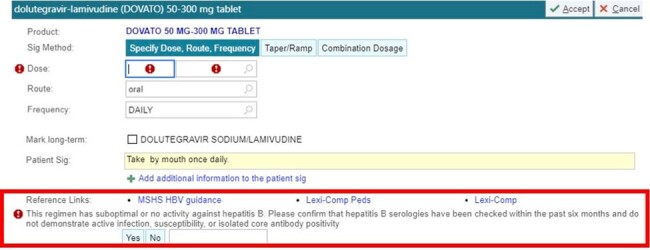
QI intervention EHR flag for 2DR orders

**Results:**

During the study period, there were 306 switches to 2DR (161 pre-intervention: 62% DTG-3TC, 22% DTG-RPV, 16% CAB-RPV; 145 post-intervention: 54% DTG-3TC, 17% DTG-RPV, 29% CAB-RPV). After QI implementation, 103 (71%) individuals had complete HBV serologies performed within 1 year prior to 2DR switch compared with 48 (30%) pre-implementation (two-tailed p < 0.0001). Post-implementation there were fewer 2DR switches among individuals at increased risk for HBV-related events (pre-QI 12, post-QI 1; two-tailed p < 0.01).

**Table 1:**
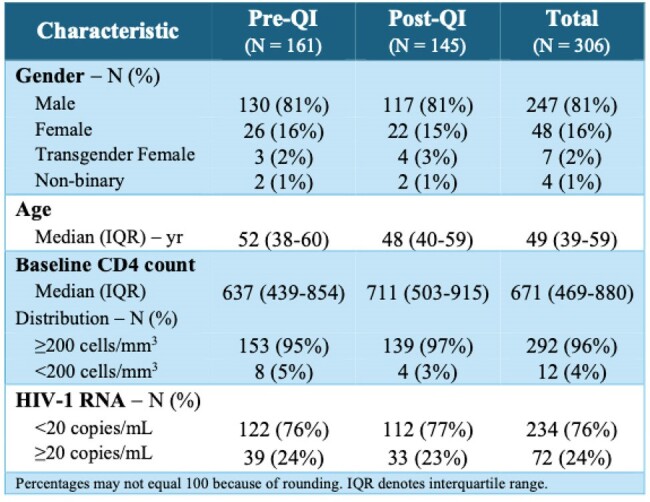
Characteristics of the participants at baseline, before and after QI implementation

**Conclusion:**

HBV infection, relapse or reactivation risk may be overlooked by clinicians when switching to 2DR for HIV. In this pilot QI study, a simple EHR-based intervention increased HBV screening prior to switch and decreased switches in those at highest risk of HBV reactivation (sAg+ or isolated cAb+). With the increasing uptake of 2DR, optimizing patient selection is vitally important to prevent HBV-related events.

**Table 2:**
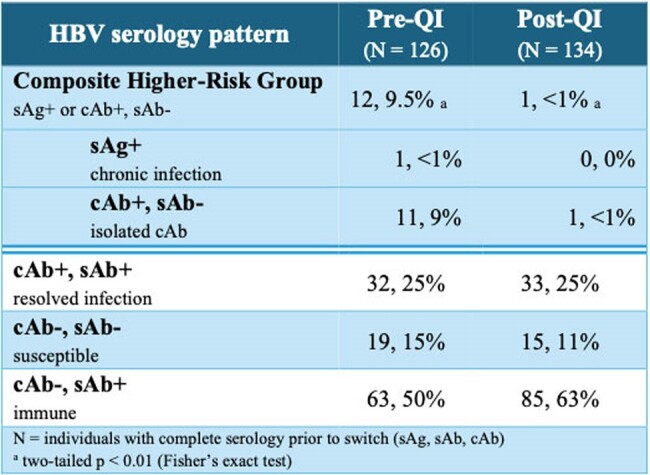
HBV serologies at time of 2DR switch, before and after QI implementation

**Disclosures:**

**Judith A. Aberg, MD**, Gilead: Grant/Research Support|GSK: Advisor/Consultant|GSK: Grant/Research Support|Janssen: Grant/Research Support|Macrogenics: Grant/Research Support|Merck: Advisor/Consultant|Merck: Grant/Research Support|Pfizer: Grant/Research Support|Regeneron: Advisor/Consultant|Regeneron: Grant/Research Support|Viiv Healthcare: Advisor/Consultant|Viiv Healthcare: Grant/Research Support **Antonio E. Urbina, MD**, Gilead: Advisor/Consultant|Merck (Any division): Advisor/Consultant|VIIV: Advisor/Consultant **Douglas Dieterich, MD**, Gilead: Advisor/Consultant|Gilead: Honoraria

